# Association between Motor Signs and Cognitive Performance in Cognitively Unimpaired Older Adults: A Cross-Sectional Study Using the NACC Database

**DOI:** 10.3390/brainsci12101365

**Published:** 2022-10-08

**Authors:** Vasileios Siokas, Ioannis Liampas, Constantine G. Lyketsos, Efthimios Dardiotis

**Affiliations:** 1 Department of Neurology, University Hospital of Larissa, Faculty of Medicine, School of Health Sciences, University of Thessaly, 41100 Larissa, Greece; 2 Department of Psychiatry and Behavioral Sciences, Johns Hopkins School of Medicine, Baltimore, MD 21205, USA

**Keywords:** cognitive performance, UPDRS, motor signs, impaired chair rise, bradykinesia

## Abstract

Aiming to examine whether specific motor signs are associated with worse performance in specific cognitive domains among cognitively unimpaired (CU) individuals, we performed a cross-sectional analysis of data from the baseline evaluations of older, CU participants from the National Alzheimer’s Coordinating Center (NACC) Uniform Data Set. In total, 8149 CU (≥60 years) participants were included. Of these, 905 individuals scored ≥ 2 on at least one of the motor domains of the Unified Parkinson’s Disease Rating Scale part III (UPDRSIII). Cognitively impaired individuals, participants with psychiatric disorders and/or under treatment with antipsychotic, anxiolytic, sedative or hypnotic agents were excluded. Nine motor signs were examined: hypophonia, masked facies, resting tremor, action/postural tremor, rigidity, bradykinesia, impaired chair rise, impaired posture/gait and postural instability. Their association with performance on episodic memory, semantic memory, language, attention, processing speed or executive function was assessed using crude and adjusted linear regression models. Individuals with impaired chair rise had worse episodic memory, semantic memory, processing speed and executive function, while those with bradykinesia had worse language, processing speed and executive function. Sensitivity analyses, by excluding participants with cerebrovascular disease or PD, or other Parkinsonism, produced similar results with the exception of the relationship between bradykinesia and language performance.

## 1. Introduction

Individuals diagnosed with dementia often present neurologic signs and cognitive symptoms years before the diagnosis of the syndrome [1,2]. Motor signs are common clinical features of dementia syndromes [3,4]. The presence of motor signs in patients with Alzheimer’s disease (AD) has been associated with worse cognitive decline and faster disease progression [5,6,7,8,9]. Apart from AD, motor signs have been associated with poorer cognitive performance in patients with schizophrenia [10], while specific characteristics of the motor signs may predict cognitive decline in patients with Parkinson’s disease (PD) [11,12].

Motor signs and cognitive performance appear to be parallel manifestations of underlying brain disease. Studies regarding trajectories of motor and cognitive performance in the general population found that decline of motor and cognitive function may vary, and that one may predate the other [13,14,15]. Moreover, motor change and cognitive decline share common neuropathology (e.g., AD pathology in motor-related brain regions, neuroinflammation, degeneration of the basal forebrain cholinergic system, Lewy body pathology) and risk factors (e.g., APOE ε4 carriage, odor identification, medical factors, psychosocial) [16,17,18].

Of note, motor signs may be useful clinical markers for better differentiation between dementia subtypes [19]. However, motor signs are under-recorded in clinical practice [3]. Deeper knowledge regarding associations between motor signs and cognitive performance could shed more light onto the underlying implicated mechanisms, and further increase awareness of the importance of their identification and clinical recording [3].

A methodological limitation of the current literature regarding motor signs and cognitive performance has been focus on specific dementia phenotypes [20]. Moreover, studies evaluating the possible role of motor signs in cognitive performance focus on specific neuropsychological tests without covering a broad range of cognitive performance. Consequently, it cannot be estimated with high certainty, which specific cognitive domains (and to what extend) are associated with specific motor signs [12,21,22,23].

To address these gaps, we cross-sectionally analyzed data from the Uniform Data Set (UDS), a standardized set of prospectively collected data from multiple Alzheimer’s Disease Research Centers (ADRCs) across the United States. The aim of this study was to examine associations between motor signs and cognitive performance in cognitively unimpaired (CU) older (≥60 years) adults. Using the Unified Parkinson’s Disease Rating Scale part III (UPDRSIII) we examined whether specific motor signs are associated with worse cognitive performance in specific domains: episodic memory, language, attention, semantic memory, processing speed and executive function.

## 2. Materials and Methods

### 2.1. General Information

The National Alzheimer’s Coordinating Center (NACC) has administrated the UDS initiative since 2005. UDS is a freely available repository (https://naccdata.org/, accessed on 1 July 2022) containing data (clinical, genetic and neuropathological) from all National Institute on Aging-funded ADRCs across the United States. Local Institutional Review Boards supervise each ADRC. All participants (or representatives) granted informed consent before inclusion. Details about the UDS have been previously extensively described [24]. Clinical personnel, trained physicians and other personnel used a common, standardized evaluation protocol and collected data from in-person visits or via telephone calls. Annually, follow-up assessments of the participants were also performed.

### 2.2. Eligibility Criteria for Participation

We included data from each participant’s first NACC visit (baseline). The recruiting period was between September 2005 through December 2021 and involved participants from 43 ADRCs aged ≥60 years. A participant was eligible for inclusion as CU because of the absence of dementia, MCI or cognitive impairment not MCI, based on physician diagnosis. For the majority of participants, the cognitive assessments were performed by interdisciplinary consensus teams. Data regarding personal history, psychosocial function and neuropsychological performance were considered in order for a diagnosis to be established. Standard clinical criteria were applied for dementia syndromes and MCI clinical diagnosis [25,26,27,28,29], while cognitively impaired participants who did not meet criteria for dementia/MCI were diagnosed as cognitively impaired—not MCI.

### 2.3. Exclusion Criteria

The following exclusions were applied: (1) treatment with an FDA-approved medication for AD; (2) presence of a psychiatric disorder diagnosed on clinical basis; (3) treatment with hypnotic, antipsychotic, sedative or anxiolytic drugs. The rational for these exclusions was to avoid potential confounding effects of these conditions on cognitive performance [30,31,32].

### 2.4. Measurement of Motor Symptoms

UPDRSIII was used to assess the presence or absence of motor signs. The UPDRSIII consists of 27 subitems rated during a neurological examination. For clinical purposes, we grouped the 27 subitems of the UPDRSIII into 9 signs [19] as follows: (1) hypophonia (single item); (2) masked facies (single item); (3) resting tremor (combined 5 items regarding tremor at rest in the face/lips/chin and 4 extremities); (4) action/postural tremor (combined 2 items regarding tremor at rest in the hands); (5) rigidity (combined 5 items regarding rigidity in the neck and 4 extremities); (6) bradykinesia (combined 9 items: bilateral finger tapping, hand movements, rapid alternating movements of the hands, leg agility and body bradykinesia); (7) impaired chair rise (single item); (8) impaired posture/gait (combined 2 items: posture and gait); and (9) postural instability (single item).

Each motor sign was graded as absent (score < 2) or present (score ≥ 2). The rational for this cutoff is as follows: (1) this level of severity is more likely to be noted by the average clinician [5]; and (2) a score of 1 is suggestive of a very mild motor change that could be observed with normal aging [19].

Then, we created a dichotomous categorical variable, such that participants were said to have an abnormal UPDRSIII if they scored ≥2 in at least one of the 9 created signs. Otherwise, they were considered to have a normal UPDRSIII. Similarly, a dichotomous motor sign variable was created for each of the 9 signs, with participants considered to have a motor domain if they scored ≥2 in at least one of the items of each domain.

### 2.5. Measurement of Cognitive Performance

The following cognitive domains were considered: (1) episodic memory (delayed and immediate recall) evaluated with the Logical Memory Test (Story A) from the Wechsler Memory Scale—Revised (WMS-R) [33]; (2) language on total word production summing animal and vegetable fluency tasks [34]; (3) semantic memory based on the 30-item version of the Boston Naming Test (BNT-30) [35]; (4) attention assessed on the Digit Span Test (DST, forward and backward conditions) from the WMS-R [33]; (5) processing speed on the Trail Making Test—Part A (TMT-A) and (6) executive function on the Trail Making Test—Part B (TMT-B) [36]. Details about the scoring of the above-mentioned tests have been reported [24]. In brief, episodic memory (the sum of items recalled in the delayed recall (0–25 total items retrieved) and immediate (0–25 total items retrieved) tasks), language (the sum of word production in the vegetable and animal 1-min category fluency tasks), semantic memory (BNT-30 (0–30 items recalled)), attention (the sum of the longest sequences in DST backward (0–7 digits) and forward (0–8 digits) conditions), processing speed (total time in TMT-A (0–150 s)) and, finally, executive function (total time in TMT-B (0–300 s)).

### 2.6. Covariates

The following covariates were considered for every participant, when possible, as they may confound the relationship between motor signs and cognitive performance: age in years at the time of the first evaluation, education in years of formal schooling (as continuous variables); sex, race; history of the following: cardiovascular disease (including heart attack, cardiac arrest, congestive heart failure or heart surgical procedures (coronal angioplasty, endarterectomy, stent or cardiac bypass procedure)), cerebrovascular disease (including transient ischemic attack or stroke), Parkinson’s disease, other Parkinsonian disorder, traumatic brain injury (TBI), thyroid disease, history of epileptic seizures, vitamin B12 deficiency, alcohol or other substance abuse and current use of antidepressant agents, as categorical variables.

### 2.7. Statistical Analysis

Continuous variables are described with means and standard deviations (SD). Values for categorical variables are expressed as total number (n) as well as percentage of the total (%). Differences in demographic and clinical characteristics between the participants with normal UPDRSIII and those with abnormal UPDRSIII were compared with independent *t*-tests for the continuous variables, and Pearson’s chi-squared tests for categorical ones.

As decline of motor and cognitive function may vary, and one may predate the other, we considered in the analysis the motor signs as the independent variables and the performance in cognitive domains as the dependent variables, for presentation purposes only. Consequently, we estimated linear regression models to examine associations between the 9 motor signs and cognitive performance on the 6 neuropsychological domains. We adjusted analyses for the covariates that significantly differed between those normal and those with abnormal UPDRSIII and motor signs.

Sensitivity analyses were performed using the same approach by excluding CU participants with at least one of the following: (1) PD, (2) other Parkinsonian disorders and (3) cerebrovascular disease.

Correction for multiple comparisons was made with the conservative Bonferroni method. We divided the conventional threshold of α = 0.05 with the number of the comparisons performed in the main analysis. We examine the association between 9 motor signs and 6 cognitive domains (54 comparisons in total), leading to a statistically significant threshold of *p* < 0.05/54 = 0.000925. The statistical analysis was performed using the IBM SPSS Statistics Software Version 26 (Chicago, IL, USA).

## 3. Results

### 3.1. Baseline Characteristics

The initial sample consisted of 17,605 CU participants. After the exclusion of participants with age lower that than 60 years old, those receiving FDA-approved medication for AD, antipsychotic, hypnotic, sedative or anxiolytic agents, those with a clinician-based diagnosis of a psychiatric disease and participants without UPDRSIII assessment, 8149 participants (n = 7244 with normal UPDRSIII and n = 905 with abnormal UPDRSIII) were included in the analysis. A flowchart of participant selection is in Figure S1. The baseline characteristics of the included participants and comparison between those with normal and those with abnormal UPDRSIII are in Table 1.

The number of the participants per motor sign included in the analysis for each cognitive domain are in Table S1.

The group with abnormal UPDRS ratings was older and less educated. The frequency of cardiovascular disease, cerebrovascular disease, PD, other Parkinsonian disorder, TBI, history of seizures and use of antidepressants was higher in the abnormal UPDRSIII group. Participants with abnormal UPDRS performed worse in all 6 cognitive domains assessed.

### 3.2. Main Analysis

#### 3.2.1. Motor Signs and Episodic Memory

Bradykinesia, impaired rise and postural instability were associated with worse cognitive performance in episodic memory in unadjusted analysis. The statistically significant association between impaired chair rise and worse episodic memory remained in adjusted regression (Table 2).

#### 3.2.2. Motor Signs and Language

Bradykinesia, impaired rise, impaired posture/gait and postural instability were associated with worse cognitive performance in language in unadjusted analysis. The statistically significant association between bradykinesia and worse language performance was maintained in adjusted linear regression (Table 3).

#### 3.2.3. Motor Signs and Attention

Bradykinesia, impaired rise, impaired posture/gait and postural instability were associated with worse cognitive performance in attention in unadjusted analysis. None of these associations were maintained in adjusted linear regression (Table 4).

#### 3.2.4. Motor Signs and Semantic Memory

Bradykinesia, impaired rise, impaired posture/gait and postural instability were associated with worse cognitive performance in semantic memory in unadjusted analysis. The statistically significant association between impaired chair rise and worse semantic memory was maintained in adjusted linear regression (Table 5).

#### 3.2.5. Motor Signs and Processing Speed

All motor signs were associated with worse cognitive performance in processing speed in unadjusted analysis. The statistically significant associations for bradykinesia and impaired chair rise were maintained in adjusted linear regression (Table 6).

#### 3.2.6. Motor Signs and Executive Function

Hypophonia, rigidity, bradykinesia, impaired rise, impaired posture/gait and postural instability were associated with worse cognitive performance in executive function in unadjusted analysis. The statistically significant associations for bradykinesia and impaired chair rise were maintained in adjusted linear regression (Table 7).

### 3.3. Sensitivity Analysis

The number of the participants per motor signs included in the sensitivity analysis for each cognitive domain are in Table S2. Overall, adjusted linear regression models (after excluding participants with at least one of the following: (1) PD, (2) other Parkinsonian disorders and (3) cerebrovascular disease) produced similar results with the main full adjusted analyses, with the exception of the association between bradykinesia and worse performance in language.

In summary, impaired chair rise was associated with worse cognitive performance in episodic memory, semantic memory, processing speed and executive function, while bradykinesia was associated with worse cognitive performance in processing speed and executive function. Results presenting the adjusted linear regression analysis for the association between motor signs and cognitive performance are presented at Table S3.

## 4. Discussion

We examined associations between 9 motor signs and cognitive performance in 6 domains among CU older adults. Our analyses revealed that participants with impaired chair rise or bradykinesia had worse cognitive performance in several domains: impaired chair rise was associated with worse episodic memory, semantic memory, processing speed and executive function, while bradykinesia was associated with worse language, processing speed and executive function. These results persisted after adjustment and sensitivity analyses, with the exception of the relationship between bradykinesia and language performance. Consequently, impaired chair rise and bradykinesia may be candidates as clinical indicators for worse cognitive performance among CU older adults.

Motor signs have emerged as possible predictors of dementia risk as well as markers of different types of dementia [19]. Bradykinesia has been associated with increased risk for dementia in patients with PD and in patients with MCI [37,38]. Impaired chair rise has been associated with reduced ability to perform activities of daily living in PD patients [39]. Moreover, greater gait impairment may predict dementia, and postural instability has been associated with increased dementia risk in MCI and CU individuals [38,40,41].

From a pathophysiological view, there are shared common pathologies between motor and cognitive functions, such as vascular risk factors, inflammation and neurodegeneration [42]. Moreover, motor signs in patients with autosomal dominant AD are related to the amount of fibrillar amyloid-β in the basal ganglia a [18]. Moreover, persons with AD have more β-amyloid deposition in both brain and muscle fibers compared with individuals without dementia [43]. However, the exact pathophysiological processes underlying the relationship between cortical amyloid deposit and motor performance remains elusive [44].

The current study has several strengths. First, we included of large number of clinically well-characterized participants assessed using a rigorous protocol. Second, we accounted for many potential cofounding variables. Third, motor signs were clinically assessed using the UPDRSIII [45]. Finally, the major findings were impervious to adjustment and sensitivity analyses, suggesting a generalization independent of clinical conditions that are related to motor disability.

We also acknowledge study limitations. First, this is a cross-sectional study with all inherent limitations [46]. Second, we dichotomized motor signs on UPDRSIII, based on whether a participant scored ≥2 in at least one of the items of each domain. Consequently, the analysis did not account overall motor function as a continuous variable. Despite the rationale behind the use ≥2 as a cut-off for the presence of motor sign, we bear in mind that some motor signs (e.g., resting tremor and the impaired gait) may be considered abnormal even with a value equal to 1, regardless of age [19]. Finally, despite the fact that we performed adjusted analysis for many potential cofounding variables, the possibility of the latent effect of other potential co-founders (e.g., subjective cognitive decline, history of infections or frailty) cannot totally be excluded.

From a clinical perspective, the associations between motor signs and cognitive performance raise interesting issues. Motor signs can be easily assessed by the majority of physicians independent of specialty, even in primary care. As such they may serve as a low-cost alternative to higher-cost indicators of worse cognitive performance [47,48] allowing for earlier detection of CU individuals with low cognitive performance and the opportunity to apply personalized cognitive training and rehabilitation. Therefore, longitudinal research with additional correction for age-related disorders associated with motor signs in older persons is needed. Moreover, combined cognitive and motor training seems to improve cognitive and motor performances in AD and PD [49,50]. Towards this direction, future studies should examine the clinical applicability of our finding, by measuring the effects of combined cognitive with physical rehabilitation in improving cognitive performance in CU individuals with motor signs.

## Figures and Tables

**Table 1:** Baseline differences between cognitively unimpaired individuals with normal and with abnormal UPDRSIII.

Variable	Normal UPDRSIII (N = 7244)	Abnormal UPDRSIII (N = 905)	*p*-Value
Age in years	73.19 ± 7.68	78.91 ± 8.65	**<0.001**
Formal education in years	15.62 ± 3.05	15.06 ± 3.37	**<0.001**
Sex (male/female)	2578 (35.6%)/4666 (64.4%)	353 (39.0%)/552 (61.0%)	**0.043**
Race (White/African American/American Indian or Alaska Native/Native Hawaiian or Pacific Islander/Asian/Other)	5763 (79.6%)/1214 (16.8%)/31 (0.4%)/5 (0.1%)/173 (2.4%)/37 (0.5%)	714 (78.9%)/151 (16.7%)/6 (0.7%)/1 (0.1%)/23 (2.5%)/6 (0.7%)	**<0.001**
Cardiovascular disease (No/Yes)	6454 (89.1%)/785 (10.8%)	730 (80.7%)/171 (18.9%)	**<0.001**
Cerebrovascular disease (No/Yes)	6793 (93.8%)/417 (5.8%)	779 (86.1%)/117 (12.9%)	**<0.001**
Parkinson’s disease (No/Yes)	7231 (99.8%)/12 (0.2%)	824 (91.0%)/76 (8.4%)	**<0.001**
Other Parkinsonian disorder (No/Yes)	7226 (99.8%)/10 (0.1%)	868 (95.9%)/33 (3.6%)	**<0.001**
Traumatic brain injury (No/Yes)	6570 (90.7%)/623 (8.6%)	801 (88.5%)/98 (10.8%)	**0.026**
History of seizures (No/Yes)	7130 (98.4%)/103 (1.4%)	881 (97.3%)/21 (2.3%)	**0.032**
Thyroid disease (No/Yes)	5849 (80.7%)/1357 (18.7%)	716 (79.1%)/181 (20.0%)	0.332
B12 deficiency (No/Yes)	6866 (94.8%)/252 (3.5%)	843 (93.1%)/41 (4.5%)	0.101
Alcohol abuse (No/Yes)	7049 (97.3%)/187 (2.6%)	870 (96.1%)/32 (3.5%)	0.092
Other substance abuse (No/Yes)	7171 (99.0%)/57 (0.8%)	898 (99.2%)/5 (0.6%)	0.444
Use of antidepressants (No/Yes)	6500 (89.7%)/744 (10.3%)	784 (86.6%)/121 (13.4%)	**0.004**
Episodic memory (sum of items recalled in the immediate and delayed recall tasks)	25.48 ± 7.62	23.44 ± 8.32	**<0.001**
Language (sum of word production in the animals and vegetables lists)	34.56 ± 8.43	31.07 ± 8.35	**<0.001**
Semantic Memory (BNT-30)	27.02 ± 3.31	25.73 ± 4.42	**<0.001**
Attention (sum of longest sequences in DST forward and backward conditions)	11.57 ± 1.99	11.04 ± 2.11	**<0.001**
Processing speed (TMT-A seconds)	34.71 ± 15.42	45.22 ± 23.00	**<0.001**
Executive function (TMT-B seconds)	91.47 ± 49.64	120.67 ± 63.36	**<0.001**

UPDRS: Unified Parkinson’s Disease Rating Scale; BNT: Boston naming test; DST: digit span test; TMT-A: trails making test—part A; TMT-B: trails making test—part B; n = number of participants with available data per parameter. Abnormal UPDRS: if at least one of the UPDRS motor signs with value ≥ 2. Statistically significant values are given in bold.

**Table 2:** Linear regression analysis for association between motor signs and episodic memory.

	Unadjusted				Adjusted			
Motor Signs	B	95% CI		*p*-Value	B	95% CI		*p*-Value
		LL	UL			LL	UL	
Hypophonia	−3.366	−6.237	−0.496	0.022	−1.607	−4.605	1.391	0.293
Masked Faces	−2.917	−4.932	−0.902	0.005	0.031	−2.373	2.436	0.98
Resting tremor	−1.728	−3.217	−0.239	0.023	−0.747	−2.378	0.884	0.369
Action/Postural Tremor	−1.078	−2.376	0.22	0.103	0.151	−1.178	1.48	0.824
Rigidity	−0.973	−2.165	0.219	0.11	1.003	−0.321	2.326	0.138
Bradykinesia	−2.435	−3.304	−1.565	**<0.000001**	−1.169	−2.172	−0.167	0.022
Impaired Chair Rise	−4.044	−5.013	−3.076	**<0.000001**	−2.092	−3.155	−1.03	**0.000114**
Impaired Posture/Gait	−1.673	−2.718	−0.627	0.002	1.421	0.254	2.587	0.017
Postural Instability	−2.237	−3.383	−1.091	**0.000131**	−0.345	−1.528	0.838	0.568

B, unstandardized correlation coefficient; CI, confidence interval; LL, lower limit; UL, upper limit. Adjusted analysis for age, years of education, sex, race, cardiovascular disease, cerebrovascular disease, Parkinson’s disease, other Parkinsonian disorder, traumatic brain injury, history of seizures and use of antidepressants, and motor signs. Statistically significant values are given in bold.

**Table 3:** Linear regression analysis for association between motor signs and language.

	Unadjusted				Adjusted			
Motor Signs	B	95% CI		*p*-Value	B	95% CI		*p*-Value
		LL	UL			LL	UL	
Hypophonia	−4.714	−7.868	−1.56	0.003	−3.066	−6.231	0.099	0.058
Masked Faces	−2.826	−5.06	−0.591	0.013	−0.731	−3.304	1.841	0.577
Resting tremor	−1.808	−3.437	−0.179	0.03	−0.143	−1.851	1.566	0.87
Action/Postural Tremor	−2.126	−3.551	−0.701	0.003	0.227	−1.174	1.628	0.751
Rigidity	−1.92	−3.226	−0.614	0.004	0.189	−1.206	1.585	0.79
Bradykinesia	−4.191	−5.142	−3.239	**<0.000001**	−2.013	−3.068	−0.958	**0.000185**
Impaired Chair Rise	−5.198	−6.259	−4.137	**<0.000001**	−1.428	−2.55	−0.306	0.013
Impaired Posture/Gait	−3.637	−4.786	−2.487	**<0.000001**	0.872	−0.359	2.104	0.165
Postural Instability	−4.088	−5.341	−2.834	**<0.000001**	−0.632	−1.875	0.611	0.319

B, unstandardized correlation coefficient; CI, confidence interval; LL, lower limit; UL, upper limit. Adjusted analysis for age, years of education, sex, race, cardiovascular disease, cerebrovascular disease, Parkinson’s disease, other Parkinsonian disorder, traumatic brain injury, history of seizures and use of antidepressants, and motor signs. Statistically significant values are given in bold.

**Table 4:** Linear regression analysis for association between motor signs and attention.

	Unadjusted				Adjusted			
Motor Signs	B	95% CI		*p*-Value	B	95% CI		*p*-Value
		LL	UL			LL	UL	
Hypophonia	−0.418	−1.129	0.293	0.249	−0.456	−1.205	0.292	0.232
Masked Faces	0.081	−0.436	0.597	0.76	0.394	−0.231	1.02	0.217
Resting tremor	−0.243	−0.629	0.144	0.218	−0.15	−0.577	0.277	0.491
Action/Postural Tremor	−0.152	−0.488	0.185	0.378	0.131	−0.219	0.481	0.462
Rigidity	−0.238	−0.547	0.07	0.129	−0.019	−0.367	0.329	0.915
Bradykinesia	−0.698	−0.923	−0.473	**<0.000001**	−0.408	−0.673	−0.144	0.002
Impaired Chair Rise	−0.86	−1.112	−0.607	**<0.000001**	−0.22	−0.501	0.061	0.125
Impaired Posture/Gait	−0.554	−0.828	−0.28	**0.000074**	0.111	−0.199	0.422	0.483
Postural Instability	−0.822	−1.118	−0.526	**<0.000001**	−0.314	−0.625	−0.003	0.048

B, unstandardized correlation coefficient; CI, confidence interval; LL, lower limit; UL, upper limit. Adjusted analysis for age, years of education, sex, race, cardiovascular disease, cerebrovascular disease, Parkinson’s disease, other Parkinsonian disorder, traumatic brain injury, history of seizures and use of antidepressants, and motor signs. Statistically significant values are given in bold.

**Table 5:** Linear regression analysis for association between motor signs and semantic memory.

	Unadjusted				Adjusted			
Motor Signs	B	95% CI		*p*-Value	B	95% CI		*p*-Value
		LL	UL			LL	UL	
Hypophonia	−0.491	−1.762	0.779	0.448	−0.945	−2.236	0.347	0.152
Masked Faces	0.792	−0.108	1.691	0.085	0.803	−0.245	1.852	0.133
Resting tremor	−0.099	−0.77	0.572	0.772	−0.437	−1.149	0.275	0.229
Action/Postural Tremor	0.14	−0.442	0.723	0.636	0.614	0.035	1.193	0.038
Rigidity	−0.365	−0.904	0.173	0.184	−0.115	−0.696	0.467	0.699
Bradykinesia	−1.237	−1.63	−0.844	**<0.000001**	−0.557	−0.996	−0.118	0.013
Impaired Chair Rise	−2.38	−2.816	−1.944	**<0.000001**	−0.849	−1.316	−0.383	**0.000358**
Impaired Posture/Gait	−1.87	−2.342	−1.398	**<0.000001**	−0.569	−1.082	−0.056	0.03
Postural Instability	−1.63	−2.147	−1.112	**<0.000001**	−0.397	−0.919	0.124	0.136

B, unstandardized correlation coefficient; CI, confidence interval; LL, lower limit; UL, upper limit. Adjusted analysis for age, years of education, sex, race, cardiovascular disease, cerebrovascular disease, Parkinson’s disease, other Parkinsonian disorder, traumatic brain injury, history of seizures and use of antidepressants, and motor signs. Statistically significant values are given in bold.

**Table 6:** Linear regression analysis for association between motor signs and processing speed.

	Unadjusted				Adjusted			
Motor Signs	B	95% CI		*p*-Value	B	95% CI		*p*-Value
		LL	UL			LL	UL	
Hypophonia	12.738	6.73	18.746	**0.000033**	7.57	1.539	13.601	0.014
Masked Faces	8.432	4.065	12.799	**0.000155**	2.93	−2.044	7.904	0.248
Resting tremor	7.95	4.724	11.175	**0.000001**	4.649	1.284	8.014	0.007
Action/Postural Tremor	6.013	3.172	8.853	**0.000034**	1.247	−1.541	4.034	0.381
Rigidity	6.653	4.054	9.252	**0.000001**	0.19	−2.574	2.953	0.893
Bradykinesia	12.762	10.869	14.656	**<0.000001**	6.194	4.077	8.31	**<0.000001**
Impaired Chair Rise	16.402	14.301	18.502	**<0.000001**	6.28	4.029	8.53	**<0.000001**
Impaired Posture/Gait	14.88	12.582	17.179	**<0.000001**	4.179	1.708	6.649	0.001
Postural Instability	12.14	9.631	14.65	**<0.000001**	1.547	−0.957	4.052	0.226

B, unstandardized correlation coefficient; CI, confidence interval; LL, lower limit; UL, upper limit. Adjusted analysis for age, years of education, sex, race, cardiovascular disease, cerebrovascular disease, Parkinson’s disease, other Parkinsonian disorder, traumatic brain injury, history of seizures and use of antidepressants, and motor signs. Statistically significant values are given in bold.

**Table 7:** Linear regression analysis for association between motor signs and executive function.

	Unadjusted				Adjusted			
Motor Signs	B	95% CI		*p*-Value	B	95% CI		*p*-Value
		LL	UL			LL	UL	
Hypophonia	36.536	17.82	55.252	**0.000131**	24.894	6.724	43.064	0.007
Masked Faces	16.547	2.819	30.276	0.018	4.372	−10.827	19.571	0.573
Resting tremor	10.991	0.797	21.185	0.035	1.647	−8.629	11.922	0.753
Action/Postural Tremor	12.117	3.232	21.003	0.008	1.013	−7.419	9.445	0.814
Rigidity	14.963	6.745	23.182	**0.00036**	−2.234	−10.683	6.216	0.604
Bradykinesia	32.351	26.337	38.366	**<0.000001**	12.692	6.214	19.169	**0.000124**
Impaired Chair Rise	50.443	43.73	57.156	**<0.000001**	17.87	10.933	24.807	**<0.000001**
Impaired Posture/Gait	40.862	33.565	48.159	**<0.000001**	10.033	2.479	17.586	0.009
Postural Instability	37.584	29.627	45.542	**<0.000001**	10.41	2.76	18.06	0.008

B, unstandardized correlation coefficient; CI, confidence interval; LL, lower limit; UL, upper limit. Adjusted analysis for age, years of education, sex, race, cardiovascular disease, cerebrovascular disease, Parkinson’s disease, other Parkinsonian disorder, traumatic brain injury, history of seizures and use of antidepressants, and motor signs. Statistically significant values are given in bold.

## Data Availability

For further information on access to the NACC database, please contact NACC (contact details can be found at https://naccdata.org/, accessed on 1 July 2022).

## Supplementary Materials

The following supporting information can be downloaded at: https://www.mdpi.com/article/10.3390/brainsci12101365/s1, Figure S1: Flowchart of participant selection; Tables S1–S3: (Table S1: Number of the subjects per 9 created motor signs and cognitive domains; Table S2: The number of the subjects per 9 created motor signs and cognitive domains, after excluding subjects with cerebrovascular disease or Parkinson’s disease or other Parkinsonian disorder; Table S3: Sensitivity analysis with linear regression, for association between motor signs (that significantly associated in the main analyses) and performance in cognitive domains, after excluding subjects with cerebrovascular disease or Parkinson’s disease or other Parkinsonian disorder.)
