# Supplementary material for: Association between Motor Signs and Cognitive Performance in Cognitively Unimpaired Older Adults: A Cross-Sectional Study Using the NACC Database
Source: Brain Sci. 2022 Oct 8;12(10):1365. doi: 10.3390/brainsci12101365 (PMC9599814; doi:10.3390/brainsci12101365)
Supplement: Supplementary file 1 [file brainsci-12-01365-s001.zip › Table S1 to Table S3.pdf]

**Table S1**. Number of the subjects per 9 created motor signs and cognitive domains						
	Episodic memory	Language	Attention	Semantic memory	Processing speed	Executive function
Hypophonia						
Yes	28	28	31	29	30	30
No	7913	7933	7932	7925	7890	7806
Masked Faces						
Yes	57	56	59	58	57	56
No	7885	7906	7905	7897	7864	7781
Resting tremor						
Yes	105	106	106	105	105	102
No	7835	7854	7857	7849	7815	7734
Action/Postural Tremor						
Yes	139	139	140	140	136	135
No	7801	7821	7823	7814	7784	7701
Rigidity						
Yes	165	166	168	164	162	158
No	7768	7787	7788	7783	7751	7672
Bradykinesia						
Yes	315	316	320	313	306	295
No	7579	7596	7595	7594	7570	7497
Impaired Chair Rise						
Yes	271	252	252	249	244	232
No	7659	7676	7679	7674	7647	7576
Impaired Posture/Gait						
Yes	216	215	213	213	205	198
No	7692	7711	7714	7707	7682	7606
Postural Instability						
Yes	179	180	181	176	172	166
No	7688	7705	7705	7705	7678	7601

**Table S2**. The number of the subjects per 9 created motor signs and cognitive domains, after excluding subjects with cerebrovascular disease or Parkinson's disease or other parkinsonian disorder						
	Episodic memory	Language	Attention	Semantic memory	Processing speed	Executive function
Hypophonia						
Yes	NT	NT	NT	NT	NT	NT
No	NT	NT	NT	NT	NT	NT
Masked Faces						
Yes	NT	NT	NT	NT	NT	NT
No	NT	NT	NT	NT	NT	NT
Resting tremor						
Yes	NT	NT	NT	NT	NT	NT
No	NT	NT	NT	NT	NT	NT
Action/Postural Tremor						
Yes	NT	NT	NT	NT	NT	NT
No	NT	NT	NT	NT	NT	NT
Rigidity						
Yes	NT	NT	NT	NT	NT	NT
No	NT	NT	NT	NT	NT	NT
Bradykinesia						
Yes	NT	203	NT	NT	198	190
No	NT	7035	NT	NT	7013	6948
Impaired Chair Rise						
Yes	201	NT	NT	200	196	187
No	7028	NT	NT	7045	7024	6962
Impaired Posture/Gait						
Yes	NT	NT	NT	NT	156	NT
No	NT	NT	NT	NT	7064	NT
Postural Instability						
Yes	NT	NT	NT	NT	NT	NT
No	NT	NT	NT	NT	NT	NT
NT, non-tested in regression models						

Table S3. Sensitivity analysis with linear regression, for association between motor signs (that significantly associated in the main analyses) and performance in cognitive domains, after excluding subjects with cerebrovascular disease or Parkinson's disease or other parkinsonian disorder

	B	95% CI		P-value
		LL	UL	
**Episodic memory**				
Impaired Chair Rise	-2.204	-3.373	-1.036	**0.000219**
**Language**				
Bradykinesia	-2.108	-3.322	-0.894	0.001
**Semantic memory**				
Impaired Chair Rise	-0.93	-1.443	-0.418	**0.000373**
**Processing speed**				
Bradykinesia	5.197	2.847	7.548	**0.000015**
Impaired Chair Rise	6.194	3.787	8.601	**<0.000001**
**Executive function**				
Bradykinesia	13.582	6.27	20.895	**0.000273**
Impaired Chair Rise	20.384	12.844	27.924	**<0.000001**

B, unstandardized correlation coefficient; CI, confidence interval; LL, lower limit; UL, upper limit

Adjusted analysis for age, years of education, sex, race, cardiovascular disease, traumatic brain injury, history of seizures, use of antidepressants and motor signs

Statistically significant values are given in bold
