# Supplementary figures and images for: Association between Motor Signs and Cognitive Performance in Cognitively Unimpaired Older Adults: A Cross-Sectional Study Using the NACC Database
Source: Brain Sci. 2022 Oct 8;12(10):1365. doi: 10.3390/brainsci12101365 (PMC9599814; doi:10.3390/brainsci12101365)

**Figure S1** Flowchart of participant selection

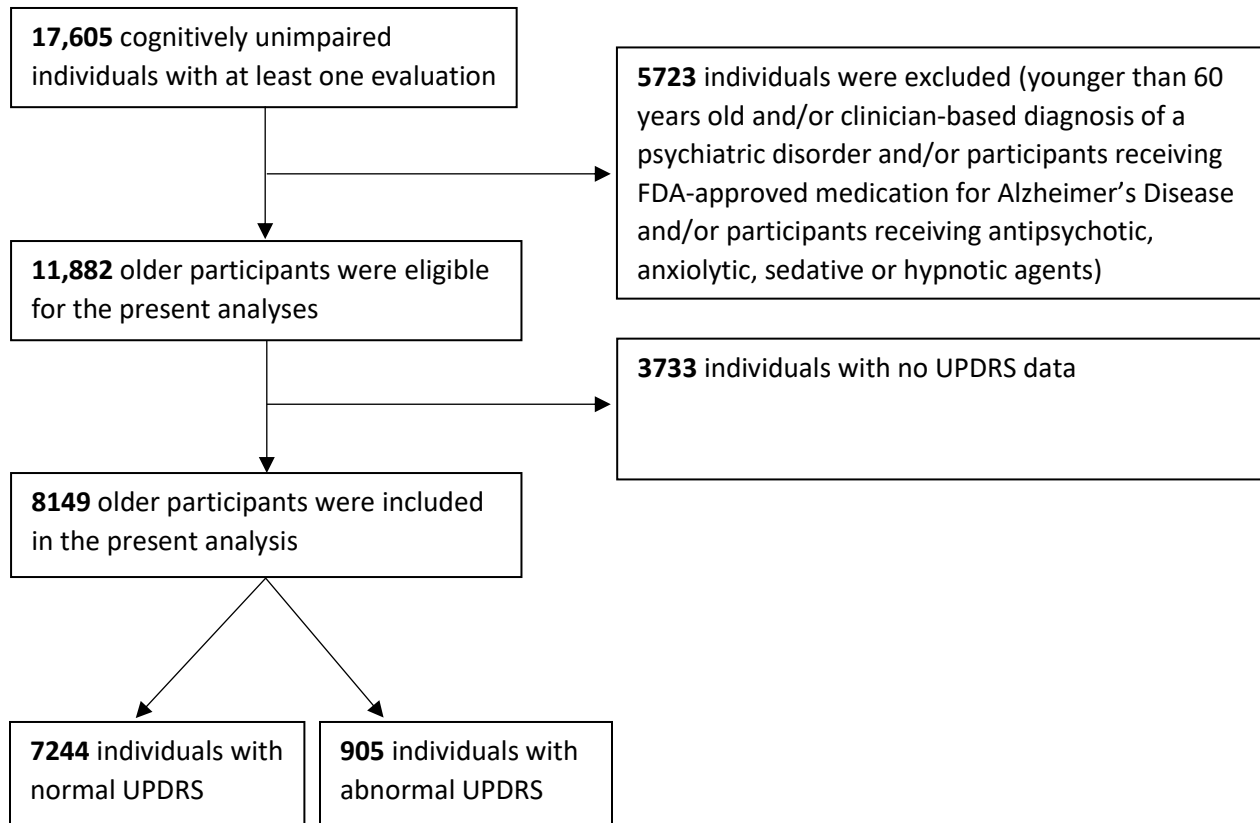

Supplement: Supplementary file 1 [file brainsci-12-01365-s001.zip › Figure S1.pdf]
